# Should we splint traumatised primary teeth?

**DOI:** 10.1038/s41432-023-00914-3

**Published:** 2023-07-11

**Authors:** Chris Deery

**Affiliations:** School of Clinical Dentistry, University of Sheffield, Sheffield, UK

**Keywords:** Dental trauma, Paediatric dentistry

## Abstract

**Design:**

Systematic review.

**Review question:**

Does splinting of traumatised primary teeth improve clinical outcomes?

**Case selection:**

Clinical studies published after 2003 reporting trauma (luxation, root fracture or alveolar fracture) to primary teeth, with a minimum follow-up of 6 months, were eligible for inclusion. Case reports were excluded, but case series were included. Studies reporting the outcome of splinting following avulsion injuries were excluded, as current guidance does not recommend re-implantation of teeth for these injuries.

**Data analysis:**

Two researchers independently assessed the risk of bias in the included studies, with a third researcher resolving any disagreements. The same two independent researchers conducted a quality assessment of the included studies.

**Results:**

Three retrospective studies met the inclusion criteria. Only one of these studies had a control group. High success rates were reported for the management of teeth with root fractures. A benefit for splinting teeth with lateral luxation was not identified. No alveolar fractures were included.

**Conclusions:**

This review suggests that the outcome of the management of root fractures in primary teeth may benefit from flexible splinting. However, the evidence base is low.

## A Commentary on

**Dos Santos Fernandez M, Schuch H S, Araújo A B, Goettems M L**

Splinting in the management of dental trauma in the primary dentition: a systematic review. *Eur Arch Paediatr Dent* 2023; **24:** 167–175.

**GRADE Rating:** 

## Commentary

This review followed the preferred Reporting Items for Systematic Reviews and Meta-Analyses (PRISMA) statement checklist^1^. Seven electronic databases were searched (MEDLINE [via PubMed], Web of Science, Scopus, Scielo, Embase, EBSCO, and Cochrane Library). A search for grey literature was also performed on Google Scholar. In addition, the reference list of the studies included in this systematic review was further manually assessed, as well as all issues published between 2013–2023 of the Journal of Dentistry, Dental Traumatology and International Journal of Paediatric Dentistry. It is perhaps disappointing that other paediatric dentistry journals were not searched, but it is unlikely many if any, further studies would have been identified.

As recognised by the authors of this systematic review, the strength of evidence provided by three non-randomised retrospective studies, only one of which had a control group, is low^2–4^. The studies all had a quality score of <5 on the Newcastle-Ottawa Scale, representing “fair quality”^5^. Generally, the number of teeth being followed when broken down by type of injury was relatively small.

A further source of bias is that it seems likely that the more severe injuries are the ones most likely to be splinted. It is recognised that with any dental alveolar trauma, the severity of the injury is more significant than the treatment in terms of predicting outcome, with less severe injuries having better outcomes^3^. This problem with studies would only be overcome by randomisation. This issue of a lack of randomised trials is seen across the dental trauma literature.

The conclusion that splinting leads to better outcomes in teeth with intra-alveolar root fractures is based on the results of two of the studies, reporting the results for 69 teeth^2,4^. In the study by Cho et al. (2018), splinted teeth were 4.67 times more likely to be retained successfully than non-splinted teeth. However, the sample was small (*n* = 28)^4^. It should be noted that 14/16 teeth in the study by Kim et al. (2012) exfoliated due to rapid resorption within ten months of the trauma^2^. Although this may seem disappointing, retaining the tooth for some time after the trauma and the avoidance of an extraction that may otherwise have been necessary is potentially beneficial. It should also be noted that there was no disturbance observed in the eruption of the permanent successor.

Overall, for luxated teeth, whether splinted or not, the prognosis is poor, with less than 40% being retained for the length of follow-up of these studies^3,4^.

The results of this systematic review support current guidelines for the management of intra-alveolar root fractures and that splinting with a flexible splint is a potential benefit^6^. However, the benefit of splinting for the management of laterally luxated teeth was not identified.

Practice points

- Splinting of primary teeth with intra-alveolar root fractures may lead to better outcomes.
- Splinting of primary teeth with luxation injuries can be considered, particularly if it improves patient comfort, but whether splinting improves outcomes is uncertain.
